# The Role of Biomarkers and Clinical Prediction Tools in the Diagnosis of Acute Aortic Syndromes: A Literature-Based Review

**DOI:** 10.3390/medicina61091551

**Published:** 2025-08-29

**Authors:** Giulia Pignataro, Alice Scafetta, Donatella De Luca, Laura Simeoli, Andrea Piccioni, Veronica Ojetti, Francesco Franceschi, Marcello Candelli

**Affiliations:** 1 Emergency, Anesthesiological and Reanimation Sciences Department, Fondazione Policlinico Universitario A. Gemelli-IRCCS of Rome, 00168 Rome, Italy; alice.scafetta01@icatt.it (A.S.); donatella.deluca01@icatt.it (D.D.L.); laura.simeoli01@icatt.it (L.S.); andrea.piccioni@policlinicogemelli.it (A.P.); francesco.franceschi@policlinicogemelli.it (F.F.); marcello.candelli@policlinicogemelli.it (M.C.); 2 Department of Internal Medicine, UniCamillus International Medical University of Rome, 00131 Rome, Italy; veronica.ojetti@unicamillus.org

**Keywords:** acute aortic syndrome, aortic dissection, biomarker, score, diagnosis

## Abstract

Acute aortic syndromes (AAS) include a spectrum of life-threatening conditions that pose considerable diagnostic challenges, particularly in emergency care settings. Clinical scores and circulating biomarkers have become essential in improving diagnostic accuracy, risk stratification, and guiding clinical decision-making. Tools such as the Aortic Dissection Detection Risk Score (ADD-RS) and the AORTAs score offer structured methods for identifying patients at elevated risk; however, their diagnostic performance can be further enhanced through integration with biomarker testing and imaging modalities. Biomarkers including D-dimer, NT-proBNP, cardiac troponins, and novel candidates such as soluble ST2 (sST2) and matrix metalloproteinase-8 and 9 (MMP-8, MMP-9), have demonstrated potential in refining diagnostic and prognostic assessments with an outstanding sensibility. ADAMTS-1 and ADAMTS-4 appear to have the best diagnostic accuracy, whereas certain non-coding DNAs (miR-15a) achieve an exceptionally high negative predictive value. These biomarkers reflect key underlying mechanisms such as inflammation, oxidative stress, and vascular injury, offering valuable insights into disease severity and progression. However, limitations related to specificity, inter-cohort variability, and assay standardization currently hinder their widespread clinical adoption. Further validation through large-scale, multi-center studies is essential to establish their role within integrated diagnostic pathways.

## 1. Introduction

Acute aortic syndromes (AASs) are life-threatening conditions affecting the thoracic aorta. These syndromes include acute aortic dissection (AAD), intramural hematoma (IMH), and penetrating aortic ulcer (PAU). Among them, AAD is the most common thoracic aortic emergency [1,2]. AAD occurs when a tear in the aortic intima permits blood to penetrate the aortic media, separating the intima from the media and/or adventitia and creating a false lumen [3]. AASs have an annual incidence of 5–15 cases per 100,000 individuals and frequently present with nonspecific symptoms, posing significant challenges for timely diagnosis. These conditions are associated with high morbidity and mortality rates [4]. In cases of AAD, the mortality risk increases by 1–2% with each hour that passes after symptom onset during the first 24 h [5]. For this reason, the Collaborative Acute Aortic Syndrome Project (CAASP) registry examined the principal determinants of diagnostic delay, which were found to include mode of presentation, underlying comorbidities, hypertension, atypical clinical manifestations, initial assessment in non-specialist centers, the requirement for inter-hospital transfer, non-white ethnicity, and socioeconomic disparities. A systematic analysis of these factors is essential to improve patient prognosis, as it may inform strategies aimed at earlier recognition, optimization of referral pathways, and equitable access to specialist care [6] The diagnosis of AAS in the emergency room (ER) is particularly challenging due to its rarity, the wide spectrum of nonspecific clinical presentations, and its overlap with more common conditions such as acute coronary syndromes or pulmonary embolism. To address these challenges, numerous studies have focused on developing scoring systems and biomarkers to reduce both misdiagnosis and unnecessary testing for AAS. These tools aim to provide clinicians with a systematic and efficient approach to screen patients, enabling early and accurate diagnosis [3].

## 2. Methods

**Figure 1:**
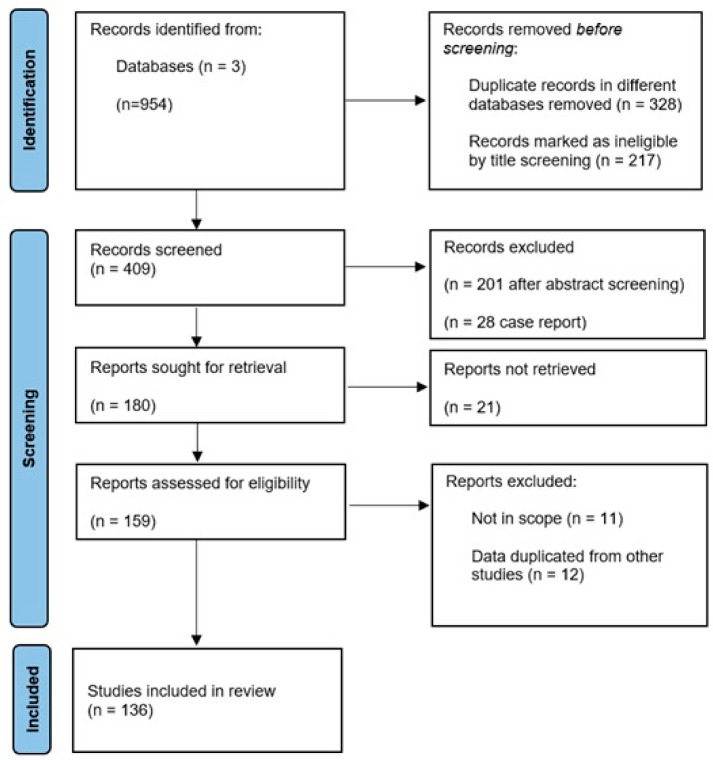
Prisma diagram flow.

This review included papers published in the last 30 years (from 1995 to 2025) about the clinical score and biomarkers used to diagnosis AASs. We searched literature reviews, observational studies (case-control, cross-sectional), retrospective and prospective studies, and clinical trials. We extracted data on the basis of period of research, title, abstract, and type of study. We searched on PubMed^®^, Web of Science^®^, and Scopus^®^. The principal words we searched are as follows: score AND acute aortic syndrome OR aorta aneurisms OR aorta dissection; biomarkers AND acute aortic syndrome OR aorta aneurisms OR aorta dissection; diagnosis or prognosis AND acute aortic syndrome OR aorta aneurisms OR aorta dissection. Duplicates from the various databases were removed, and a screening of all the articles found, first based on their titles and then on their abstracts, was performed. Negative studies, case reports, letters to the editor, comments, and any type of correspondence were excluded. All works not relevant to the purpose of the review and articles that were not accessible were excluded (Figure 1).

## 3. Classification and Clinical Presentation of Acute Aortic Syndromes

AAD can be classified based on anatomical features or the timing of symptom onset. The two primary systems for anatomical classification are the De Bakey system and the Stanford classification (Table 1).

Type A dissections are approximately twice as common as Type B dissections and carry a significantly higher risk of fatality without prompt intervention [6]. Regarding temporal classification, a dissection is considered chronic if it begins two or more weeks before clinical presentation; otherwise, it is classified as acute. Alternatively, some authors adopt a more detailed time frame, defining a dissection as acute if symptoms began within 0 to 7 days, subacute for an onset between 8 to 30 days, and chronic if symptoms started more than 30 days prior [7,8].

Recently, a new classification based on type of dissection, location of the tear of the primary entry, and malperfusion (TEM) has been proposed and is increasingly used by clinicians and researchers [9]. The TEM classification is based upon the traditional Stanford system but introduces a third category, the “non-A non-B” type, which includes dissections that do not fit the classic type A or type B definitions. It evaluates three dimensions: T specifies the type of dissection; E identifies the location of the initial intimal tear, distinguishing whether it originates in the ascending aorta, the aortic arch, or the descending aorta; and M assesses malperfusion syndromes, which are further subclassified according to the arterial territories involved—coronary arteries, supra-aortic trunks, visceral and renal arteries, or lower limb arteries—and whether these perfusion deficits are clinically evident or detectable only through imaging. This comprehensive, multidimensional framework provides a more nuanced characterization of aortic dissections, supports tailored therapeutic strategies, enhances prognostic accuracy, and facilitates precise interdisciplinary communication [10].

Diagnosing AASs can be particularly challenging due to several factors. Definitive diagnostic tests may not always be readily available in the ER, and the variable clinical presentations of AAS often lead to missed, delayed, or incorrect diagnoses in up to 40% of cases [11]. Alarmingly, some AAS cases are only identified during post-mortem examinations [12,13]. This diagnostic complexity is further compounded by the absence of typical symptoms in many patients, which can divert clinicians toward incorrect diagnostic pathways. Additionally, limited access to advanced imaging modalities in certain ER settings poses a significant barrier. Emergency doctors may also hesitate to order imaging studies for patients deemed to have low or intermediate risk for AAS, further delaying diagnosis [14,15,16]. These challenges become particularly pronounced in cases with atypical presentations, such as painless malperfusion phenomena, dyspnea caused by heart failure or pleural effusion, elevated troponin levels, electrocardiogram findings resembling acute coronary syndrome, limb ischemia, or abdominal pain. The most common presentation of AAD in the ER is an elderly patient with hypertension and sudden chest pain. Differentiating AAD from the more prevalent acute coronary syndrome is critical, as the two conditions share overlapping signs and symptoms. AAD pain is typically described as sudden, severe, and “sharp” or “shooting” [17,18], often affecting both the chest and back, in contrast to the gradual onset of angina. Maintaining a high index of suspicion is essential when evaluating patients for AAS, especially given the variability of symptoms based on the dissection’s location. Type A dissections most commonly present with chest or back pain, while Type B dissections may cause abdominal pain if the dissection extends into the abdominal region. Syncope can also be a presenting symptom of AAD and is associated with worse outcomes [19].

## 4. Diagnostic Imaging Techniques for Acute Aortic Syndromes

Currently, no rapid, accurate, and easily accessible diagnostic test exists for AAD, making definitive imaging essential for diagnosis. Common imaging modalities include transthoracic echocardiography (TTE), computed tomography angiography (CTA), transesophageal echocardiography (TEE), and magnetic resonance imaging (MRI). TTE, while less reliable for visualizing the distal aorta, remains valuable for assessing acute aortic syndromes and related complications, such as aortic regurgitation, left ventricular dysfunction, or pericardial effusion (which may suggest a rupture). It is often employed as an initial bedside tool, particularly in unstable patients [19]. CTA is the first-line imaging modality in most cases due to its high sensitivity and specificity, rapid image acquisition, and widespread availability. It provides detailed visualization of the aorta, identifying key features such as the intimal flap, true and false lumens, aortic dilatation, intramural hematoma, and complications like contrast extravasation (indicating rupture) or branch vessel involvement [19]. TEE is often preferred in hemodynamically unstable patients or when CTA is unavailable. TEE provides high-resolution, real-time images of the aortic root, ascending aorta, and descending thoracic aorta, making it particularly effective for diagnosing proximal dissections (Type A) and associated complications such as aortic regurgitation or cardiac tamponade [19]. While MRI is highly accurate, it is less commonly used in acute settings due to its limited availability and longer acquisition times. However, MRI is excellent for visualizing branch vessel involvement and offers superior soft tissue contrast, making it a preferred option in select cases [20]. In specific cases, and particularly for the assessment of type B aortic dissection, the utility of 4D flow MRI has recently been described. This technique has proven valuable in evaluating prognosis and guiding surgical indications for this type of aneurysm, especially in instances where urgent surgery is not immediately indicated [21]. Similarly, for type B aortic aneurysms, 8F-NaF PET/CT has been shown in a recent study to be a promising tool for risk stratification when combined with clinical parameters and biomarkers. Functional assessment of the aorta demonstrated advantages over purely anatomical imaging in evaluating the risk of aneurysm progression [22,23]. The choice of diagnostic modality depends on the level of clinical suspicion, but each test carries the potential for logistical delays in patient management [2,3,4,5,6,7,8,9].

## 5. Artificial Intelligence and AAS

AI holds substantial promise in the diagnosis and management of AAS. By leveraging advanced computational approaches, AI can support the diagnostic and prognostic process through complementary strategies, including the analysis of gene expression to identify potential biomarkers, the rapid interpretation of imaging studies, and the integration of heterogeneous clinical data. One of the most innovative applications is the ability to screen thousands of genes implicated in AAS pathophysiology and identify candidate biomarkers with potential diagnostic or prognostic relevance. Examples include:

- Lactylation-related genes: *PGK1, HMGA1* [24]
- Cellular senescence-related genes: *CHEK1, FOXM1, BRCA1* [25]
- Disulfidptosis-related genes: *INF2, CD2AP, CAPZB* [26]
- Immune-related markers: *CXCL1, ITGA5, PTX3, TIMP1* [27]
- Pyroptosis-related genes: *CASP4, MLKL, PECAM1, HDAC6* [28]
- Ferroptosis-related genes: *CA9, HMOX1, IL6, CDKN1A, HIF1A, MYC* [29]

These molecular candidates, has been identified through machine learning techniques and provide valuable insights into the pathophysiology of AAS, although their clinical utility has not yet been confirmed and no prospective studies are available to support their routine use. Another promising field of applicatio is imaging, where AI algorithms can assist clinicians in detecting subtle features of aortic disease and expedite decision-making in critical settings. For instance, AI-driven CT image analysis has shown high diagnostic performance with pooled sensitivity and specificity of 94% and 88% and an AUC of 0.97, although clinical adoption is limited by heterogeneity and risk of bias [30], while convolutional neural network-based pipelines for abdominal CT scans have achieved near-perfect sensitivity in detecting abdominal aortic dissections, streamlining intrahospital workflows [31]. Furthermore, AI enables the integration of vast and heterogeneous clinical, laboratory, and instrumental data into predictive models with high accuracy. Multimodal deep learning approaches combining ECG features, laboratory results, and demographic information achieved an AUC up to 0.98 in differentiating acute Stanford type A dissection from acute myocardial infarction [32], while models using ECG data alone, such as the AI-Aortic-Dissection-ECG (AADE) score, reached an accuracy of 0.93 and correlated with mortality risk [33]. Beyond diagnostics, AI also supports risk stratification and triage: machine learning algorithms, such as the SuperLearner ensemble model, have been shown to improve prehospital triage of suspected AAS, reducing undertriage to 1% while maintaining acceptable overtriage and supporting timely referral to specialist centers [34]. Despite these advances, it is important to emphasize that none of these biomarkers or computational approaches have yet undergone robust clinical validation, and their impact on real-world outcomes remains unproven. Future research should focus on prospective multicenter studies that integrate molecular, imaging, and computational findings to ensure reproducibility, safety, and effectiveness before AI can be fully implemented in routine clinical practice.

## 6. Diagnostic Scores in Acute Aortic Syndromes

Diagnostic scores are invaluable tools in clinical practice, offering structured approaches to risk stratification and decision-making across a wide range of medical conditions. By integrating clinical, laboratory, and imaging data, these tools help clinicians identify high-risk patients, prioritize diagnostic testing, and optimize the use of healthcare resources. In acute settings, where time-sensitive decisions are essential, diagnostic scores play a crucial role in achieving a balance between sensitivity and specificity. They facilitate the accurate identification of life-threatening conditions while minimizing unnecessary investigations, thereby enhancing efficiency and patient outcomes. This section explores the application and utility of diagnostic scores in the context of AASs, emphasizing their strengths, limitations, and emerging developments.

### 6.1. Aortic Dissection Detection Risk Score (ADD-RS)

The ADD-RS, proposed in the 2010 guidelines by Hiratzka et al. [19], is a fundamental tool for assessing the likelihood of AAS. This system stratifies patients into low, intermediate, and high-risk categories based on clinical features, including predisposing conditions, pain characteristics, and findings from the physical examination. A point is assigned for the presence of predisposing conditions such as Marfan syndrome, a family history of aortic disease, or known aortic valve disease; pain characteristics, including sudden onset, tearing or ripping quality, and maximum intensity at onset; and clinical findings such as evidence of perfusion deficits, new neurological symptoms, or hypotension. This structured approach ensures a systematic evaluation and allows for the rapid and accurate identification of high-risk patients. The simplicity of the ADD-RS makes it particularly valuable in high-pressure environments like the emergency room. Its effectiveness has been validated in multiple studies, including the well-known IRAD registry [35], leading to its widespread adoption in clinical practice. However, the ADD-RS is not without its limitations. While its simplicity enhances usability, the tool demonstrates moderate sensitivity when used alone, particularly in patients considered low risk. Moreover, it relies heavily on the clinician’s judgment, which introduces variability in interpretation across different observers. Another limitation is its lack of biomarkers or imaging data, which could significantly improve its predictive accuracy, especially in borderline or ambiguous cases. In conclusion, while the ADD-RS is an invaluable tool for the initial evaluation of patients with suspected AAS, its limitations highlight the need for complementary diagnostic methods to ensure a more accurate and comprehensive risk assessment (Table 2).

### 6.2. Aortic Dissection Detection Risk Score (ADD-RS) Combined with D-dimer

Nazerian et al. [36] demonstrated that combining the ADD-RS with D-dimer testing significantly improves diagnostic accuracy for AAS, particularly for ruling out the condition in low- to intermediate-risk patients. D-dimer, a biomarker of fibrinolytic activity, is elevated in nearly all cases of acute aortic dissection due to the underlying thrombotic processes. The ADD-RS with D-dimer testing showed a sensitivity of 98% and a negative predictive value (NPV) exceeding 99%, results corroborated by subsequent research [37]. This approach is cost-effective and non-invasive, reducing unnecessary imaging when clinical suspicion is low. Elevated D-dimer levels are nonspecific and may result from other conditions such as pulmonary embolism, malignancy, or trauma. Therefore, while a low D-dimer is reliable for ruling out AAS, elevated levels require further confirmatory testing.

The structured approach of the ADD-RS was refined in the 2014 ESC guidelines [44], which introduced a flowchart to bridge the gap between scoring systems and clinical application. The process begins with identifying high-risk conditions, pain characteristics, and examination findings. High-risk patients (ADD-RS ≥ 2) are recommended for immediate advanced imaging of the neck, chest, abdomen, and pelvis. Lower-risk patients (ADD-RS < 2) undergo additional evaluations including ECG to rule out alternative diagnoses such as STEMI, along with D-dimer testing, chest X-ray, and point-of-care ultrasound (POCUS) where available. This algorithm ensures rapid and comprehensive assessment, minimizing missed cases of AAS while avoiding excessive use of imaging. The 2024 ESC guidelines present an updated visual flowchart integrating ADD-RS scoring with diagnostic pathways, supporting clear communication and streamlined emergency workflows. Moreover, a recent study demonstrated that the integration of bedside imaging techniques (point-of-care ultrasound, POCUS) with the ADD score and D-dimer testing achieved a sensitivity of 100% (97.9–100) for the diagnosis of AAS, with a specificity of 52.3% (49.9–54.6) or 58.8% (56.5–61.6), depending on the D-dimer cutoff applied (500 ng/mL or age-adjusted, respectively). This combined approach showed better performance than the use of D-dimer and ADD score alone [45].

### 6.3. Simplified Acute Aortic Dissection (SAAD) Score

The SAAD score, introduced by Morello et al. in 2021 [43], is a risk stratification tool developed to enhance the diagnosis of AAS, particularly in elderly patients. By combining clinical assessment with age-adjusted D-dimer thresholds, the SAAD score addresses a significant limitation of the ADD-RS: the high rate of false positives in older populations due to the natural increase in D-dimer levels with age. The clinical probability component of the SAAD score builds on the framework of the ADD-RS, incorporating predisposing conditions, pain characteristics, and abnormal findings on physical examination. This ensures that the score remains straightforward and accessible, serving as an effective starting point for risk stratification. The inclusion of age-adjusted D-dimer thresholds adds an important layer of refinement. For patients aged 50 years or older, the D-dimer cutoff is calculated as age multiplied by 10 ng/mL (when values are expressed in ng/mL), while the cutoff for younger patients remains at 500 ng/mL. This adjustment significantly reduces false positives in elderly patients, optimizing the use of diagnostic resources and avoiding unnecessary imaging. The SAAD score offers several advantages that make it a valuable addition to clinical practice. Its simplicity mirrors the structured design of the ADD-RS, ensuring ease of use and allowing seamless integration into everyday workflows. This is particularly beneficial in emergency settings, where rapid and accurate decision-making is critical. One of the score’s most notable strengths is its enhanced accuracy in elderly patients, achieved using age-adjusted D-dimer thresholds. This feature not only minimizes overtesting but also maintains a high level of diagnostic precision. The SAAD score has undergone rigorous validation in multicenter studies, demonstrating a sensitivity of 97.6% and a specificity of 74.3% for detecting AAS [23]. These findings underscore its reliability and effectiveness as a diagnostic tool, providing clinicians with confidence in its application. By combining ease of use with evidence-based accuracy, the SAAD score represents a significant advancement in the diagnosis and management of AAS, particularly in populations where traditional approaches may fall short.

### 6.4. Aortic Occlusion Risk Tool for Acute Syndromes (AORTAs) Score

The AORTAs score was introduced as a comprehensive tool designed to stratify the risk of AAS by integrating clinical, laboratory, and imaging data. This scoring system assigns weighted points to key risk factors, including patient history, physical findings, D-dimer levels, and initial imaging results, providing a holistic approach to diagnosis. Patient history plays a crucial role in the AORTAs score, with emphasis on high-risk conditions such as a bicuspid aortic valve or a history of prior aortic surgery. Clinical findings, including pulse deficits, neurological symptoms, and signs of shock, further refine the risk assessment. Laboratory data, particularly elevated D-dimer levels above age-adjusted thresholds, add another critical dimension to the score. Imaging features, such as mediastinal widening on chest X-rays or abnormal findings on bedside ultrasound, are also incorporated, ensuring a robust and multimodal evaluation process. The AORTAs score has demonstrated remarkable diagnostic accuracy. Both retrospective and prospective validations report a sensitivity exceeding 95% and a specificity of approximately 85% [43]. By integrating data from multiple sources, the score is particularly effective in refining risk stratification for intermediate-risk patients. This adaptability makes it invaluable in cases where the clinical presentation is ambiguous, offering a more nuanced and reliable assessment. One of the key strengths of the AORTAs score lies in its ability to combine imaging findings with clinical and laboratory data. Weighted points are assigned for specific imaging features, such as mediastinal widening on chest X-rays or abnormalities detected through bedside echocardiography. This comprehensive approach enables the early identification of high-risk patients, including those who might not exhibit classic symptoms, ensuring timely intervention. However, the AORTAs score is not without its limitations. Its reliance on imaging and laboratory results can hinder immediate bedside application, especially in resource-limited settings where access to advanced diagnostic tools may be restricted. Despite these challenges, the score represents a significant advancement in risk assessment for AAS. By addressing the limitations of other diagnostic tools, the AORTAs score provides a sophisticated, evidence-based framework for evaluating patients. Its multimodal design is particularly advantageous in emergency settings, where rapid and accurate decision-making is essential (Table 2).

### 6.5. Comparative Analysis: ADD-RS vs. SAAD vs. AORTAs

Several authors, including Nazerian et al. [36] and Bima et al. [46], have examined the comparative utility of the three primary diagnostic scores for AAS: ADD-RS, SAAD, and AORTAs. Their findings highlight that while all three tools are valuable for risk assessment, their effectiveness varies depending on the clinical context and resource availability. The ADD-RS and SAAD scores are particularly advantageous due to their simplicity and ease of use, making them ideal for rapid risk stratification in emergency settings where swift decision-making is critical. However, these tools primarily rely on clinical evaluation, which can occasionally lead to missed high-risk cases, especially in patients with atypical presentations. In contrast, the AORTAs score provides a more detailed and nuanced assessment by integrating imaging findings with clinical and laboratory data. This multimodal approach is particularly useful for evaluating patients with intermediate risk or ambiguous clinical features, as it offers greater diagnostic precision. Nonetheless, its application is often limited by the need for advanced imaging modalities, which may not be readily accessible in resource-constrained settings. Ultimately, the choice of risk assessment tool should be tailored to the specific clinical scenario, balancing the need for diagnostic accuracy with the practical constraints of the healthcare environment [47].

### 6.6. Other Diagnostic Scores

Ohle et al. (2023) [42] introduced the Canadian RIPP score, a novel diagnostic tool that integrates clinical, radiographic, and laboratory data to stratify the risk of AAS. Preliminary studies have demonstrated its strong diagnostic performance, with a sensitivity of 96% and a specificity of 82%. Similarly, Song et al. [48] explored the utility of combining the ADD-RS with chest X-ray findings, such as mediastinal widening, to enhance pre-imaging risk stratification. These emerging tools address critical gaps in current diagnostic strategies, particularly in cases where traditional scores may produce inconclusive results. The RIPP score’s approach to combining clinical and imaging data aligns closely with the principles underlying the AORTAs score, offering a more comprehensive evaluation of risk, and highlights its potential for broader applicability across diverse patient populations and clinical settings. The RIPP score may significantly enhance the accuracy and efficiency of AAS diagnosis, improving patient outcomes (Table 2).

### 6.7. An Integrative Approach: Coagulation, Laboratory Markers, and Scores

The integration of coagulation markers and laboratory indices is increasingly recognized as a valuable advancement in the diagnosis of AAS. Dong et al. (2017) [49] highlighted the strong correlation between elevated D-dimer levels and AAS, while also emphasizing the diagnostic potential of other markers such as fibrin degradation products (FDP) and platelet counts. When combined with clinical scoring systems, these markers significantly enhance diagnostic accuracy. For example, studies have demonstrated that incorporating FDP into the SAAD or AORTAs scores can markedly improve both sensitivity and specificity, thus increasing the overall diagnostic yield. Similarly, Gorla et al. (2017) [21] demonstrated the efficacy of integrating the ADD-RS score with D-dimer levels and imaging findings. This combined approach ensures an optimal balance between sensitivity and specificity, supporting more efficient resource utilization in emergency settings. Notably, the AORTAs score has shown promise when paired with biomarkers like D-dimer. Using elevated D-dimer levels as a trigger for advanced imaging in patients stratified by the AORTAs score allows clinicians to achieve a high diagnostic yield while avoiding unnecessary testing. Looking forward, these integrative strategies represent a significant step toward personalized, data-driven diagnostic pathways. Larger prospective studies are essential to validate these approaches further. Additionally, the exploration of novel biomarkers such as microRNAs and inflammatory cytokines offers exciting potential. These emerging markers could not only refine diagnostic accuracy but also differentiate AAS from other conditions with similar clinical presentations, such as myocardial infarction or pulmonary embolism. Such advancements hold promise for more tailored and effective clinical decision-making in the future.

## 7. Biomarkers

Over the past decade, numerous studies have been conducted with the aim of identifying biomarkers capable of providing early signals of disease. These studies have focused on the pathophysiology and the identification of components that, either individually or in combination, could serve as indicators of early disease onset. Biomarkers, in general, can be divided into two main categories: genetic and biochemical. Genetic biomarkers are useful for early diagnosis in familial or hereditary diseases, such as hereditary connective tissue disorders, including Marfan syndrome and Ehlers–Danlos syndrome. On the other hand, biochemical biomarkers are employed during the acute phase of disease and are the focus of this work. The aim of this section is to examine the strength of the existing literature and assess how clinical practice in emergency departments (EDs) could benefit from the use of biomarkers and in what ways they might be applied [50]. Specifically, biochemical biomarkers can be classified based on the processes that generate them, such as activation of the fibrinogen-fibrinolysis system, activation of the inflammatory system, vascular matrix damage, and myocardial muscle injury. Additionally, indirect biomarkers of hypoperfusion damage can also be considered. This section will explore the most significant biomarkers, evaluating their strengths and limitations, as well as their potential applications in clinical practice.

### 7.1. D-Dimer

D-dimer is the most extensively studied biomarker in the management of AAS and is recommended in the 2014 ESC guidelines [44] for use in low-risk patients (ADD-RS < 2). As a fibrin degradation product produced during fibrinolysis when blood contacts damaged vessel surfaces, D-dimer is elevated in many other conditions, including myocardial infarction, stroke, malignancy, deep vein thrombosis, and pulmonary embolism. Its high sensitivity makes it a valuable tool for ruling out AAS in the appropriate clinical setting.

Systematic reviews and meta-analyses consistently emphasize its diagnostic value. Wren et al. [51] reported a pooled sensitivity of 96.5% (95% CI: 94.8–98%) and a specificity of 56.2% (95% CI: 48.3–63.9%) at the 500 ng/mL threshold. A 2024 meta-analysis found that combining ADD-RS ≥ 1 with D-dimer > 500 ng/mL achieved a pooled sensitivity of 93.1% (95% CI: 87.1–96.3%) and specificity of 67.1% (95% CI: 54.4–77.7%). The IRAD-Bio study confirmed that a 500 ng/mL cutoff within 24 h of symptom onset yielded a sensitivity of 97% and specificity of 59% [52]. D-dimer levels below 100 ng/mL demonstrated 100% sensitivity, while levels above 1600 ng/mL strongly supported AAS diagnosis within six hours. The ADvISED [53] study reported a sensitivity of 96.7% and specificity of 64% when combining D-dimer with ADD-RS. Chen’s meta-analysis found a pooled sensitivity of 96% (95% CI: 93–98%) and a specificity of 72% (95% CI: 59–81%), with the 500 ng/mL cutoff increasing sensitivity to 97% (95% CI: 95–99%) but reducing specificity to 53% (95% CI: 43–63%). The diagnostic odds ratio of 56.86 (95% CI: 30.87–104.72) and an area under the curve (AUC) of 0.95 (95% CI: 0.93–0.97) highlight its reliability for ruling out AAS despite limited specificity. Asha et al. [54] confirmed these conclusions, noting high sensitivity in low-risk patients but acknowledging variable specificity across populations. While D-dimer cannot independently distinguish AAS from other causes of elevation [41,55], it is most effective for excluding AAS in low-risk patients when combined with clinical scoring systems such as the ADD-RS, enabling more efficient diagnostic pathways [56].

### 7.2. Inflammatory Markers

The investigation into the role of inflammatory responses in cardiovascular diseases began with observations and experiments that demonstrated how proinflammatory cytokines can initiate and exacerbate various pathological conditions [57]. Growing scientific evidence highlights the crucial involvement of the innate immune system in cardiovascular disorders, particularly in the progression of atherosclerosis [58]. These findings suggest that specific inflammatory cytokines may act as critical biomarkers, offering the potential for earlier and more accurate diagnosis of these diseases.

#### 7.2.1. Soluble Suppression of Tumorigenesis-2 (sST2)

sST2, a member of the interleukin-1 receptor family (IL1RL-1), plays a crucial role in immune regulation and exists in two distinct forms: a transmembrane form (ST2L) and a soluble form (sST2). The soluble form, which is released into the bloodstream, functions as a decoy receptor for IL-33. Initially identified for its involvement in inflammatory processes and cardiovascular diseases, sST2 has been extensively studied in heart failure [59], where it is overexpressed in stretched cardiac muscle cells, making it a valuable biomarker for risk stratification [38]. Given these established roles, researchers have explored sST2 as a potential biomarker for acute aortic syndrome (AAS). Wang et al. [59] demonstrated significantly elevated sST2 levels in AAS patients compared to individuals with other conditions, such as acute myocardial infarction (AMI), pulmonary embolism (PE), angina, or healthy controls. At a cutoff of 34.6 ng/mL, sST2 showed superior diagnostic performance compared to D-dimer, with a sensitivity of 99.1% and specificity of 84.9%. However, these promising findings were later challenged by Morello et al. [60], who conducted a single-center prospective study involving 287 patients, 243 of whom were classified as low-risk based on the ADD-RS score. Among the 162 patients who underwent CTA, 88 were diagnosed with AAS. Median sST2 levels were higher in AAS patients (41.7 ng/mL) compared to non-AAS cases (34.6 ng/mL), and a linear correlation was observed between sST2 and D-dimer levels. In this study, D-dimer retained its diagnostic sensitivity at 95.8% and specificity at 30.7% with a cutoff of 500 ng/mL. For sST2, the optimal diagnostic cutoff was determined to be 39.8 ng/mL, but its accuracy was modest compared to D-dimer. The discrepancies between these studies may stem from differences in patient demographics and study designs. Morello’s cohort was more diverse, including older individuals and a higher proportion of male patients, who typically exhibit elevated sST2 levels [60]. Additionally, ethnic differences between Asian and European populations may have influenced the results. Variations in the assays used to measure sST2 levels could also contribute to these inconsistencies. In conclusion, while sST2 shows promise as a biomarker for AAS, its diagnostic accuracy relative to D-dimer remains uncertain. Further large-scale studies with well-defined and standardized cohorts are necessary to establish its clinical utility and reliability.

#### 7.2.2. IL-6

Interleukin-6 (IL-6) is a cytokine that plays central roles in the proliferation, differentiation, and regulation of immune cells. Its physiological and pathological functions have been extensively studied, particularly in the context of acute aortic syndromes (AAS), including AAD. Several studies [61,62,63] have consistently shown that circulating IL-6 levels are significantly elevated in patients with AAD compared to healthy controls. Moreover, within AAD cohorts, non-survivors exhibit markedly higher IL-6 levels than survivors, highlighting its potential as a prognostic marker. At a diagnostic cutoff of 18.36 pg/mL, IL-6 demonstrated a sensitivity of 87.4% and a specificity of 70.8%, making it an effective biomarker for diagnosing AAD [64]. Compared to other inflammatory markers, IL-6 has advantageous kinetics and a favorable diagnostic window: its levels rise rapidly after the onset of symptoms, peak within 24–48 h, and then gradually decline, eventually normalizing over time. This rapid response offers a diagnostic advantage in acute clinical settings. Furthermore, elevated IL-6 levels have been associated with increased disease severity and poorer prognosis [65,66]. These findings suggest that in addition to its diagnostic value, IL-6 may serve as an important prognostic marker for patients with AAD. This dual role positions IL-6 as a promising biomarker not only for diagnosing AAD but also for guiding risk stratification and management strategies.

#### 7.2.3. IL-10

Interleukin-10 (IL-10) is a key anti-inflammatory cytokine that plays a crucial role in maintaining immune homeostasis and regulating inflammatory responses. Although it has been less extensively studied compared to other biomarkers, IL-10 has shown significant promise in the context of AAD. Elevated values of serum IL-10 have been found in patients with AAD when compared to age- and sex-matched controls [67]. Moreover IL-10 levels are notably elevated in patients with AAD compared to those with other conditions, such as acute myocardial infarction (AMI), pulmonary embolism (PE), and thoracic aortic aneurysm (TAA). At a diagnostic cutoff of 20 ng/mL, IL-10 demonstrated a sensitivity of 55.0% and an impressive specificity of 98%, highlighting its potential as a reliable diagnostic marker for AD [66]. In contrast to D-dimer, which has low sensitivity and high specificity and is therefore used to rule out AD and consequently avoid invasive radiological investigations in low-risk patients, the potential of IL-10 lies in its ability to guide low-risk subjects toward further diagnostic investigations to confirm the diagnosis of AD [65]. Furthermore, IL-10 exhibits a diagnostic time window similar to that of IL-6, suggesting its active involvement in modulating the inflammatory response during the acute phase of AAD. This combination of high specificity and a favorable temporal profile positions IL-10 as a promising candidate to complement existing diagnostic tools in the management of AD.

#### 7.2.4. C-Reactive Protein (CRP)

CRP, an acute-phase reactant produced in response to various cytokines, is widely recognized as a key marker in clinical diagnosis and management of inflammatory diseases. While CRP is inherently a highly sensitive parameter, its lack of disease specificity—particularly in the context of acute aortic dissection (AD)—limits its utility as a diagnostic marker. However, extensive research has established its reliability as a prognostic indicator [68]. Elevated CRP levels, especially those exceeding 15 mg/L, have been consistently associated with a higher risk of adverse outcomes, making it a useful marker for identifying high-risk patients [69]. These findings have been further validated by recent studies. For instance, one investigation focused on in-hospital mortality among patients with type-A AD, utilizing CRP as a prognostic biomarker. The study demonstrated a sensitivity of 77% and a specificity of 72% in predicting mortality [70]. However, variability in patient demographics, comorbidities, and inflammatory responses may have influenced these results, underscoring the need for additional research to confirm CRP’s prognostic accuracy and broader clinical applicability.

### 7.3. Adiponectin

Adiponectin, a molecule from the adipokine family produced by perivascular adipose tissue, plays a critical role in regulating metabolism and maintaining energy balance. Research has shown that adiponectin, along with angiopoietin-like proteins (ANGPTL)-8 and -2, can suppress the expression of proinflammatory cytokines, such as IL-6, particularly in the context of abdominal aortic aneurysms [71]. In a study by Yang et al. [72], the combination of ANGPTL-8, D-dimer, and high-sensitivity C-reactive protein (hs-CRP) achieved a sensitivity of 98.46% and a specificity of 79.49% in diagnostic performance. Despite these promising findings, the clinical application of adiponectin as a biomarker remains under investigation.

### 7.4. White Blood Cells (WBC), Platelet Count, Neutrophil/Lymphocytes Ratio (NLR)

Certain biomarkers investigated for acute aortic syndromes (AAS) are already in clinical use for other conditions. For example, several studies have explored the potential roles of white blood cell (WBC) and platelet counts in the management of AAS. Morello et al. examined WBC counts, platelet counts, and fibrinogen levels as supplementary tools for risk assessment in patients with a low clinical probability of AAS [73]. Their findings showed that in such patients, a WBC count > 9 × 10^3^/µL, a platelet count < 200 × 10^3^/µL, and fibrinogen < 350 mg/dL were associated with a sensitivity of 95.5% and a specificity of 18.3%. Additionally, WBC > 9 × 10^3^/µL and platelet count < 200 × 10^3^/µL were identified as independent predictors of AAS 73. Sbarouni et al. highlighted the potential of the neutrophil-to-lymphocyte ratio (NLR) in facilitating earlier identification of type-A aortic dissection (AD), particularly when combined with clinical assessments and imaging [74]. Elevated NLR levels were also associated with severe inflammation and poorer prognosis, underscoring its value as a marker for risk stratification. Zhang et al. [75] studied the combination of D-dimer and NLR, finding that while D-dimer is highly sensitive and remains essential for ruling out AAS, adding NLR improves specificity and aids in differential diagnosis. Furthermore, this combination helps prioritize patients who require urgent imaging and intervention, as both biomarkers are linked to prognosis, with higher levels correlating to worse outcomes.

### 7.5. Extracellular Matrix Markers (ECM)

The extracellular matrix (ECM), composed of structural and adhesive proteins, is crucial for vascular support and function. Imbalances in ECM synthesis or degradation can compromise the integrity of the aortic wall, increasing the risk of dissection. Fragmentation of elastic fibers, a reduction in elastin, and the degradation of collagen contribute to the loss of vascular smooth muscle cells, fibrosis, and decreased arterial compliance, ultimately making the aorta more susceptible to rupture.

#### 7.5.1. Matrix Metalloproteinases (MMPs)

Matrix metalloproteinases (MMPs) are enzymes involved in the degradation of ECM components, and their role in AAS, particularly AAD, has attracted significant attention. Aortic dissection is closely associated with degenerative changes in the aortic wall, which are often linked to dysregulation of the ECM. Excessive MMP activity can weaken the aortic wall, accelerating ECM degradation and contributing to the progression of AAD. Research by Kurihara et al. [76] investigated MMP levels in patients with AAD, AMI, and chronic aortic aneurysms and healthy individuals. The study found significantly elevated levels of MMP9 in AAD patients compared to other groups, while MMP1, MMP2, MMP3, and tissue inhibitors of metalloproteinases (TIMP1) showed no significant differences. Immunohistochemical analysis revealed the presence of MMP9-positive cells and increased neutrophil infiltration in the aortic media of AAD patients. Neutrophils were identified as the primary source of MMP9. Experimental studies in murine models further demonstrated that MMP9 plays a critical role in triggering AAD from pre-existing aneurysms, with neutrophil activity varying according to the disease stage. These findings suggest that targeting neutrophil infiltration and MMP9 activity may help reduce the incidence of AAD, highlighting MMP9 as a promising biomarker for the condition. However, further research is needed to confirm its diagnostic and therapeutic relevance. In another study, Giachino et al. [77] assessed MMP8 and MMP9 levels in an emergency setting among 126 patients with suspected AAD, 52 of whom were confirmed via CT angiography. Elevated levels of MMP8 and MMP9 were observed in AAD patients compared to those with other diagnoses or uncomplicated aortic aneurysms. MMP8 demonstrated strong correlations with D-dimer, creatinine, and CRP levels. While D-dimer exhibited the highest diagnostic accuracy, with an area under the curve (AUC) of 0.87, the use of MMP8 at a specific cutoff reduced the need for secondary imaging by 5%. When combined with D-dimer at adjusted thresholds, the need for urgent imaging decreased by 20%, suggesting that MMP8 could complement D-dimer in diagnostic strategies. Despite these promising findings, further studies are required to validate the clinical utility of MMP8 and MMP9. In conclusion, these studies highlight the potential of MMP9 and MMP8 as biomarkers for AAD. MMP9 is closely tied to the pathophysiology of AAD, while MMP8 shows potential in improving diagnostic efficiency when used alongside existing markers like D-dimer. However, additional research is crucial to fully establish their roles in clinical practice.

#### 7.5.2. Soluble Elastin Fragments (sELAF)

Soluble elastin-derived peptides (sELAF) have emerged as potential biomarkers for AAS, particularly AAD. Elastin, a crucial structural protein in the aorta, provides the elasticity and resilience necessary to withstand hemodynamic stress. In AAD, the primary pathological changes involve the degradation of elastin in the media layer, driven by inflammatory cells, elastases, and metalloproteinases [78]. These processes weaken the aortic wall, and as the dissection progresses, sELAF are released into the bloodstream. Several studies have highlighted the diagnostic potential of sELAF. Shinohara et al. [79] demonstrated that sELAF levels provided a sensitivity of 68% and specificity of 96.6%, indicating its value as a diagnostic marker. Furthermore, Peng et al. [68] reported an improved sensitivity of 82.86% using a cutoff of 97.07 ng/mL. Shinohara’s study also identified a higher cutoff value of 285.4 ng/mL for detecting the presence of a false or partially open lumen in AD, yielding a sensitivity of 88.9% and specificity of 99.8%. The kinetics of sELAF are particularly promising, as levels rise within 0.7 h of symptom onset and remain stable for 48–72 h, making it a reliable marker for early diagnosis [79]. However, despite these advantages, practical limitations currently hinder its routine use in emergency settings. Further research is necessary to optimize its application and validate its clinical utility in routine practice.

#### 7.5.3. Tenascin C (TNC)

Tenascin-C (TNC) is an extracellular matrix glycoprotein that plays a critical role in tissue repair, development, and remodeling. It is responsive to mechanical stress and inflammatory signals, making it an important player in various pathological processes. Elevated levels of TNC have been associated with several cardiovascular conditions, particularly aortic aneurysms, where its concentration correlates with larger aneurysm size and greater aortic wall damage [80]. In the context of AAD, TNC levels rise rapidly within 12–24 h of symptom onset, peaking alongside markers such as high-sensitivity C-reactive protein (hs-CRP) and D-dimer. Subsequently, TNC levels gradually decrease over the following 24 h [81]. While TNC is not primarily used for diagnostic purposes, its main utility lies in its potential as a prognostic biomarker in AAD. Guo et al. [82] explored the prognostic value of TNC for in-hospital mortality in AD patients, identifying a cutoff value of 103.4 ng/mL, which yielded a sensitivity of 83.87% and specificity of 83.33%. When combined with D-dimer, the diagnostic accuracy improved significantly, achieving a sensitivity of 90.30% and a specificity of 88.46%. These findings highlight the value of calponin in prognosis, especially when paired with D-dimer for enhanced predictive accuracy.

### 7.6. Smooth Muscle Cell (SMC) Biomarkers

SMC biomarkers are molecular markers that reflect the presence, health, or activity of smooth muscle cells, particularly in both pathological and physiological contexts. These biomarkers are crucial for studying diseases associated with vascular remodeling, such as atherosclerosis, aneurysms, and AAD. SMCs play a key role in the structural integrity of blood vessels, and their behavior—such as proliferation, migration, and phenotypic modulation—can significantly impact the progression of these conditions.

#### 7.6.1. Smooth Muscle Myosin Heavy Chain (SmMHC)

SmMHC (smooth muscle myosin heavy chain) is a key protein in smooth muscle cells, essential for muscle contraction and tension maintenance. It is part of the myosin complex, which consists of two heavy chains and four light chains. The heavy chains include a globular head that interacts with actin to generate movement and a body and tail that help form thick filaments. SmMHC is released into the bloodstream during the early stages of AAD, originating from the medial layer of the aortic wall as it undergoes damage. A study by Katoh et al. [83] showed that smMHC levels were significantly elevated, reaching concentrations 5–10 times higher in patients with AD compared to the control group within the first 24 h after symptom onset. However, the biomarker’s kinetics are rapid, with levels peaking within the initial 24 h and declining sharply in the subsequent 24 h. In a study by Suzuki et al. [84], the diagnostic performance of smMHC was assessed using a cutoff of 2.5 ng/mL, which demonstrated a sensitivity of 90% and specificity of 97% within the first 12 h after symptom onset. When measured within the first 3 h, sensitivity increased to 90.9%, and specificity rose to 98%. Moreover, smMHC levels above 10 ng/mL exhibited 100% specificity for AAD. Additionally, smMHC levels were found to rise more significantly in proximal aortic dissections compared to distal ones [84]. These findings underscore smMHC as an effective biomarker for diagnosing AAD, with the added benefit of aiding in the identification of the lesion’s location.

#### 7.6.2. Calponin

Calponin is a regulatory protein associated with actin filaments in smooth muscle cells, playing a pivotal role in regulating muscle contraction by modulating the interaction between actin and myosin. It exists in three isoforms: Calponin 1 (basic), specific to smooth muscle cells; Calponin 2 (neutral), found in both smooth muscle and some non-muscle tissues; and Calponin 3 (acidic), primarily expressed in non-muscle tissues. In patients with AAD, elevated levels of both acidic and basic calponin have been detected in both proximal and distal segments of the aorta. Acidic calponin, with a cutoff value of 2.3 ng/mL, demonstrated a sensitivity of 50% and specificity of 87%. Basic calponin, with a threshold of 159 ng/mL, achieved 63% sensitivity and 73% specificity within the first six hours following symptom onset. Over a 24 h period, acidic calponin showed 58% sensitivity and 72% specificity, while basic calponin exhibited 50% sensitivity and 66% specificity. Notably, calponin levels were found to correlate with elevated troponin levels, suggesting its potential as a prognostic marker for in-hospital mortality in AD patients [85]. Calponin has shown good diagnostic accuracy when used as a single biomarker; however, its utility appears to be enhanced when integrated with easily obtainable anatomical data. In particular, the combination of elevated calponin levels with an aortic diameter greater than 40 mm has proven especially valuable. Interestingly, these measurements were performed by emergency medicine residents with limited ultrasound experience, relying on a portable echocardiography device. Despite the non-specialist setting, the combined assessment achieved excellent sensitivity and specificity in identifying AAD, suggesting that this pragmatic approach may be useful in EDs [86]. Despite these promising findings, further research is needed to fully establish the clinical utility of calponin.

### 7.7. Cardiac Markers

Cardiac markers are biomolecules, typically proteins or enzymes, released into the bloodstream following damage or stress to the heart muscle. The presence or elevated levels of these markers in the blood are used to diagnose, monitor, and assess the severity of cardiac conditions.

#### 7.7.1. N-Terminal Pro-Brain Natriuretic Peptide (NT-proBNP)

NT-proBNP is an inactive peptide released into the bloodstream when ventricular fibers stretch, often due to volume or pressure overload. It is derived from the cleavage of pro-BNP, a precursor molecule, into two components: BNP, which is biologically active and regulates fluid balance and blood pressure, and NT-proBNP, which is biologically inactive but widely used as a clinical biomarker for cardiac stress. NT-proBNP is primarily used for diagnosing and predicting outcomes in heart failure. Although numerous studies have been conducted, NT-proBNP has not shown superiority over other biomarkers in diagnosing AD. Nonetheless, research consistently indicates elevated NT-proBNP levels in AD, potentially due to arterial hypertension, a primary risk factor for the development of AD [87]. A correlation between NT-proBNP levels and aortic diameters has also been identified [88]. As a result, NT-proBNP remains a valuable marker for assessing in-hospital mortality in patients with AD and the risk of developing secondary heart failure [89].

#### 7.7.2. Troponin I and T

Cardiac troponins, proteins specific to heart tissue, play a crucial role in regulating cardiac muscle contraction. Thera are three troponin subunits: Troponin C (TnC), which binds calcium to initiate contraction; Troponin I (TnI), which inhibits the actin-myosin interaction in the absence of calcium; and Troponin T (TnT), which anchors the complex to tropomyosin. TnI and TnT are myocardium-specific and serve as reliable biomarkers for detecting cardiac damage, particularly in diagnosing AMI. In AAS, studies have shown elevated troponin levels in some patients, often due to myocardial ischemia caused by coronary artery compression or reduced perfusion. However, troponin elevations are not specific to AAS and may lead to a misdiagnosis of AMI. This underscores the need for a thorough diagnostic approach when elevated troponin levels are detected in suspected AAS cases [90]. A recent study examined the utility of the D-dimer to high-sensitivity Troponin T (D/T) ratio as a diagnostic marker to differentiate AAS from non-ST elevation myocardial infarction (NSTEMI). Incorporating this ratio into a broader diagnostic framework for acute chest pain may help minimize delays or diagnostic errors. Additionally, it provides valuable guidance in selecting the most appropriate confirmatory test, such as the ADD-RS or the thrombolysis in myocardial infarction (TIMI) risk score [91].

#### 7.7.3. Copeptin

Copeptin, a peptide derived from the pre-pro-vasopressin pro-hormone, is released alongside vasopressin and serves as a stable marker for its blood levels. Although copeptin lacks direct biological function, it reflects vasopressin activity, which regulates water balance, blood pressure, and stress responses. Elevated copeptin levels have been observed in critical conditions such as hemorrhage, sepsis, and severe illnesses. Furthermore, a study conducted in an ED showed that patients with STEMI, NSTEMI, pulmonary embolism, and AAS had higher copeptin levels compared to patients presenting to the ED with chest pain from benign causes. However, the number of patients with AAS in this study was too small to draw any conclusions [92]. Another study found that copeptin levels were higher in AAS compared to myocardial infarction and similar to other life-threatening conditions [93]. However, copeptin alone demonstrated insufficient diagnostic accuracy for confirming or excluding AAS. Furthermore, combining copeptin with D-dimer did not enhance diagnostic performance over D-dimer alone. While copeptin was not an independent predictor of mortality in AAS patients, it was associated with higher mortality rates in those with alternative diagnoses. These findings suggest that copeptin’s value lies more in reflecting disease severity in emergency settings than in serving as a specific diagnostic tool for AAS [93].

#### 7.7.4. Ischemia-Modified Albumin (IMA)

IMA, a variant of serum albumin altered by oxidative stress or ischemia, is recognized as an early biomarker of ischemic damage. Studies have shown elevated IMA levels in patients with AAD compared to healthy individuals, suggesting its potential as a marker of ischemic and oxidative stress conditions associated with AAD. However, its specificity is limited, as increased IMA levels are also observed in other ischemic or oxidative stress-related conditions [94]. Despite these limitations, IMA demonstrates significant prognostic value. Elevated levels within the first 24 h of symptom onset have been identified as an independent risk factor for in-hospital mortality, with a sensitivity of 80.6% and a specificity of 84.8% [95].

### 7.8. Lipid Metabolism-Related Markers

Lipid metabolism-related markers are molecules that reflect the state of lipid metabolism and can provide diagnostic, prognostic, or therapeutic information in different clinical conditions. In AAS, they play a marginal but potentially useful role in the diagnosis and differential diagnosis between different lesions in AD.

#### 7.8.1. Low-Density Lipoprotein (LDL)

The role of LDL in the development and progression of cardiovascular diseases, such as atherosclerosis and acute coronary syndromes (ACS), is well established. The soluble form of lectin-like oxidized LDL receptor 1 (sLOX-1) originates from the cleavage of the extracellular domain of LOX-1, found on endothelial cells, and is released into the bloodstream. Research has demonstrated that sLOX-1 levels are associated with the severity of ACS and AAS. In a study by Kobayashi et al. [96], sLOX-1 levels were significantly higher in patients with aortic dissection (AD) compared to those with ACS, achieving a sensitivity of 89.5% and a specificity of 94.3% at a cut-off value of 150 pg/mL. Despite these promising findings, further studies are required to confirm and better understand its diagnostic utility.

#### 7.8.2. Homocysteine (Hcy)

Hcy is a sulfur-containing amino acid formed as a byproduct during methionine metabolism. It serves as an intermediate compound that is typically recycled back into methionine or converted into cysteine through enzymatic reactions dependent on B vitamins, including folate, vitamin B6, and vitamin B12. The association between elevated Hcy levels and cardiovascular disease is well-established, with Hcy recognized as an independent risk factor for the development of atherosclerosis [97]. Hcy serves as a reliable biomarker in patients with genetic collagen disorders, such as Marfan syndrome [98], but does not hold the same significance in cases of sporadic AAS.

#### 7.8.3. Lysophosphatidylcholine (LPC)

LPC is a phospholipid produced by the hydrolysis of phosphatidylcholine through the enzyme phospholipase A2 (PLA2). It plays a role in the sphingomyelin-ceramide pathway, which drives pro-inflammatory, oxidative, and apoptotic processes that contribute to atherosclerosis and aging. Studies have shown a correlation between increased sphingomyelins in the blood and AAD, along with a reduction in LPC levels. Notably, decreased sphingolipids have been observed exclusively in type-A AD [99]. Although current research is insufficient to establish these molecules as diagnostic tools, LPC and sphingolipids show promise for future use in diagnosing AD and differentiating between type-A and type-B cases.

### 7.9. Non Coding Nucleic Acid (Micro and Circular RNA)

#### 7.9.1. MicroRNA

MicroRNAs (miRNAs) are small, single-stranded non-coding RNA molecules that regulate gene expression at the post-transcriptional level. They function primarily by promoting messenger RNA (mRNA) degradation or by inhibiting translation, ultimately reducing protein synthesis. Each miRNA may influence hundreds of target mRNAs, and conversely, each mRNA can be regulated by multiple miRNAs—resulting in a complex and finely tuned regulatory network. MiRNAs are remarkably stable in both tissues and body fluids such as plasma, and their high sensitivity and specificity in detection (via techniques like microarrays and real-time PCR) make them highly attractive as non-invasive biomarkers [100]. This section focuses on the role of miRNAs in aortic diseases, particularly TAAD and AAD, and their potential use in diagnostics and mechanistic research. The members of the miR-29 family are known to suppress the expression of key extracellular matrix proteins, including collagen, fibrillin-1, and elastin. Their upregulation has been linked to weakening of the aortic wall and aneurysm formation. In patients with TAAD secondary to bicuspid aortic valve (BAV), miR-29a shows differential expression—being upregulated in the concavity and downregulated in the convexity of the aorta [101,102,103]. These findings suggest a role in regional vascular remodeling in response to altered hemodynamic stress. miR-17 is involved in ECM remodeling by modulating the balance between tissue inhibitors of metalloproteinases (TIMP) and MMPs. Wu et al. [104] reported dysregulation of the miR-17 gene cluster in BAV patients, associated with decreased TIMP1/2 and increased MMP2 activity—an imbalance linked to early aortic dilation and medial degeneration. miR-143 and the miR-145 are closely associated with VSMC phenotype regulation. miR-145 promotes a contractile phenotype by enhancing expression of contractile genes, while miR-143 suppresses proliferative pathways [105,106,107]. Together, they help maintain vascular stability and control abnormal remodeling. The miR-30 family is involved in vascular development and cellular proliferation. In TAD, Liao et al. [108] found widespread dysregulation of miRNAs, including members of the miR-30 and miR-29 families. These miRNAs appear to regulate pathways related to cell adhesion and MAPK signaling, potentially contributing to collagen deposition and structural disruption in the aortic wall. Rather than relying on single markers, combining multiple miRNAs into diagnostic panels may improve accuracy. Xu et al. [109] identified a four-miRNA signature (miR-25, miR-29a, miR-155, and miR-26b) in patients with Stanford type A AAD, achieving strong diagnostic performance even in hypertensive populations. Wang et al. [110] identified four miRNAs—miR-4313, miR-933, miR-1281, and miR-123831—that were consistently upregulated in both aortic tissue and plasma of AAD patients, highlighting their potential utility in both mechanistic studies and blood-based diagnostics.

#### 7.9.2. Circular RNAs

Circular RNAs (circRNAs) are a distinct class of non-coding RNAs characterized by a covalently closed loop structure, which makes them highly stable and resistant to exonuclease-mediated degradation. Unlike linear RNAs, circRNAs lack 5′ caps and 3′ poly-A tails, giving them enhanced durability in biological samples—an attribute that makes them promising candidates for biomarker discovery. Functionally, circRNAs often act as “sponges” for miRNAs, binding them competitively and thereby modulating the expression of downstream target genes. This regulatory mechanism, known as the competing endogenous RNA (ceRNA) effect, is increasingly recognized as a key component of gene expression networks in health and disease. Tian et al. [111] conducted a study to explore circRNA expression profiles in both pathological and healthy aortic tissues. They identified circMARK3 as significantly upregulated in patients with Stanford type A aortic dissection, both in aortic tissue and in serum. Bioinformatic analyses suggested that circMARK3 may regulate the expression of the tyrosine kinase gene Fgr, which could be implicated in the pathophysiology of dissection. Given their stability and tissue-specific expression patterns, circRNAs such as circMARK3 hold strong potential as diagnostic biomarkers. In the same study, Tian et al. [111] evaluated a diagnostic model combining serum circMARK3 with miR-1273-3p. The combined panel showed improved sensitivity and specificity compared to individual markers, highlighting the value of integrating multiple ncRNA types in diagnostic strategies for AAD. Nonetheless, several limitations remain. The diagnostic performance of circMARK3 has so far been evaluated only in symptomatic patients with confirmed AAD, limiting its utility as a predictive tool for at-risk individuals. Its specificity to AAD is also unclear, as comparisons with other cardiovascular pathologies were not included. Furthermore, while other differentially expressed circRNAs were identified, most were analyzed through computational prediction without functional validation in vitro or in vivo. Despite these challenges, circRNAs represent an exciting and rapidly expanding frontier in vascular biomarker research. With further study, they may provide valuable insights into aortic disease mechanisms and open the door to novel, non-invasive diagnostic approaches. Noncoding RNAs are emerging as promising biomarkers for aortic diseases due to their stability, tissue specificity, and direct involvement in key pathological mechanisms. Several miRNAs (e.g., miR-29, miR-17, miR-145) have shown diagnostic and prognostic potential, both individually and in combination panels. CircRNAs—such as circMARK3—have also demonstrated diagnostic value, especially when used alongside miRNAs. However, clinical application remains premature: large-scale, multicenter, and standardized studies are still needed to confirm their reliability.

### 7.10. Other Biomarkers

#### 7.10.1. Polycystin-1

Polycystin-1 (PC1) is a large membrane-bound glycoprotein encoded by the PKD1 gene, with important roles in various cellular processes and in the development of organs such as the kidneys and the cardiovascular system. When functioning in conjunction with polycystin-2 (PC2), PC1 forms a calcium-permeable ion channel involved in mechanosensitive signal transduction. In addition to its role in calcium homeostasis, PC1 regulates cell–cell and cell–matrix interactions, as well as multiple intracellular signaling pathways. It also contributes to vascular homeostasis by influencing cell proliferation, differentiation, and apoptosis. Recent research has demonstrated a significant reduction in PC1 expression within vascular smooth muscle cells (VSMCs) of patients diagnosed with AAD [112]. This decrease in expression correlates with increased activation of the ERK (extracellular signal-regulated kinase) pathway—an important driver of VSMC proliferation, apoptosis, and phenotypic switching. The functional role of PKD1 has also been examined in diagnostic contexts. Peng et al. [68] reported that serum PC1 concentrations above 357.33 pg/mL were associated with an 85.7% sensitivity and a 75.6% specificity for identifying AD. Similarly, Feng et al. [113] noted that PKD1 is directly involved in the pathophysiology of AD through modulation of VSMC contraction and MAPK signaling. Their analysis found PKD1 to be significantly downregulated in AD samples, with an enrichment of MAPK-related genes, while healthy tissues showed greater activity in VSMC contraction pathways. Further studies have implicated PKD1 in the progression of TAAD via activation of pathways such as mTOR/S6K/S6 and MEK/ERK/Myc.

#### 7.10.2. Lumican

Lumican is a small leucine-rich proteoglycan (SLRP) and a structural component of the ECM. It contributes in organizing collagen fibers, maintaining ECM architecture, and mediating interactions between cells and their surrounding matrix. These functions are needed for cellular migration, proliferation, angiogenesis, inflammation, and tissue repair—processes that are particularly relevant in vascular pathology. Gu et al. [114] explored Lumican as a diagnostic biomarker for AAD. Their study assessed serum Lumican levels in AAD patients and investigated their correlation with disease severity. Using multi-slice computed tomography angiography (MSCTA) as the reference standard, the researchers observed significantly elevated serum Lumican levels in patients compared to healthy controls. At an optimal cutoff of 35.79 ng/mL, Lumican achieved a sensitivity of 88.6% and a specificity of 90.0% in diagnosing AAD. Furthermore, it appeared to offer value in stratifying dissection severity, particularly within the first 12–72 h after symptom onset. Despite these promising results, the study had several limitations common to early biomarker research, including a relatively small sample size, limited specificity against conditions that clinically mimic AAD, and a single-center design. These factors restrict the immediate applicability of Lumican in acute clinical settings. A subsequent and more comprehensive investigation by Chen et al. [115] strengthened the case for Lumican’s clinical relevance. Their study assessed Lumican expression in both plasma and aortic tissue from AAD patients and examined its functional role using a mouse model of aortic dissection induced by angiotensin II and β-aminopropionitrile. Results showed significantly elevated levels of Lumican in both human serum and aortic tissue—particularly within the tunica media. In mice, administration of recombinant Lumican reduced AAD incidence and mortality. This protective effect was associated with reduced vascular remodeling, preservation of elastic fiber structure, suppression of matrix metalloproteinase (MMP-2 and MMP-9) activity, and enhanced expression of type I collagen. These findings suggest that Lumican not only is a promising biomarker for AAD but may also be a protective, mechanistic factor in maintaining aortic wall integrity. Nonetheless, larger multicenter studies are essential to validate its diagnostic and therapeutic potential.

#### 7.10.3. Aggrecan

Aggrecan (ACAN) is a chondroitin sulfate proteoglycan in the ECM, especially critical in load-bearing tissues. Its capacity to retain water and withstand compressive stress helps maintain the structural stability of vascular and connective tissues. Recent research suggests that ACAN could serve as a biomarker for acute type A aortic dissection (ATAAD). In a study by König et al. [116], ACAN levels were measured across various patient groups, revealing markedly higher concentrations in individuals with ATAAD compared to those with thoracic aortic aneurysms, myocardial infarction, or healthy controls. These elevated serum ACAN levels remained detectable for up to 72 h after symptom onset, potentially widening the diagnostic window. Using a cutoff value of 14.3 ng/mL, ACAN showed 97% sensitivity and 81% specificity for ATAAD diagnosis. Notably, the study found no strong association between ACAN and conventional cardiac markers like CK-MB and cTnT, implying that ACAN may reflect aortic wall injury more specifically than myocardial damage. Despite these encouraging results, the study had limitations, including a small sample size, variability in sample collection timing, and its single-center design. Further research is needed to clarify the optimal timing and clinical utility of ACAN measurement in diagnostic protocols. An ongoing study by the Chinese University of Hong Kong, titled “Potential Diagnostic Biomarkers for Aortic Dissection in the Emergency Department” (ClinicalTrials.gov Identifier: NCT06065306), is currently enrolling participants. This observational trial aims to assess the diagnostic performance of desmosine, aggrecan, and D-dimer in patients evaluated within 24 h of symptom onset. The findings may help determine whether these biomarkers can accurately identify AAD during early ED presentation.

#### 7.10.4. ADAMTS

ADAMTS (A Disintegrin And Metalloproteinase with Thrombospondin Motifs) refers to a family of 19 secreted metalloproteinases that help to remodel ECM. Although they share a common catalytic mechanism, individual ADAMTS enzymes have distinct biological functions—ranging from proteoglycan degradation (e.g., ADAMTS4, ADAMTS5) and procollagen processing (e.g., ADAMTS2, 3, and 14), to regulation of coagulation (ADAMTS13) and angiogenesis. These enzymes are also involved in developmental and homeostatic processes, particularly within the vascular system. Recent studies have implicated ADAMTS family members in the pathogenesis of AAS. In a detailed review, Kemberi et al. [117] examined the specific roles of ADAMTS1, ADAMTS4, and ADAMTS5 in thoracic aortic disease. ADAMTS5 has garnered attention due to its observed reduction in plasma levels among patients with TAAD, despite an increase in tissue expression. One study found a significant inverse association between circulating ADAMTS5 levels and aortic dissection risk, suggesting that impaired proteoglycan degradation may lead to pathological ECM accumulation in the aortic media [118]. ADAMTS4 has also shown diagnostic potential. Serum levels were elevated in ATAAD patients, achieving high diagnostic performance with a reported sensitivity of 94.6% and specificity of 97.1% [119]. However, as ADAMTS4 expression can be upregulated in inflammatory conditions such as atherosclerosis—primarily due to macrophage activity—its specificity for aortic pathology may be limited. Of note, Kaufmann et al. [120] recently developed a low-molecular-weight cyclic peptide that selectively targets ADAMTS4. In a preclinical setting, this peptide was used as a molecular MRI probe to predict abdominal aortic aneurysm expansion in mice. ADAMTS1 has consistently been reported to be overexpressed in TAAD tissue samples across multiple studies [121,122,123,124,125]. Despite these encouraging results, the clinical translation of ADAMTS enzymes as biomarkers faces several challenges. Main limitations include small sample sizes, heterogeneity in detection methods (e.g., ELISA versus immunoblot), and a lack of standardized antibody validation. Furthermore, plasma concentrations of ADAMTS proteins may not reliably reflect their tissue levels, as many are sequestered within the ECM.

#### 7.10.5. Osteopontin

Osteopontin (OPN) is a multifunctional glycoprotein abundant in the extracellular matrix, where it plays diverse roles in inflammation, tissue remodeling, and oncogenesis. Its expression is regulated by a range of cytokines and growth factors, making it a responsive marker in various pathological conditions. Yuan et al. [126] investigated the expression levels of OPN in both serum and aortic tissue from patients with AAD, aortic aneurysms, and coronary artery disease and healthy controls. The study found significantly elevated serum OPN levels in patients with acute type A AAD compared to both healthy individuals and those with CAD. However, serum levels did not significantly differ between AAD and aortic aneurysm patients, suggesting that OPN elevation may reflect general aortic wall pathology rather than dissection-specific processes. At the tissue level, OPN mRNA and protein expression were markedly increased in both AAD and aneurysm samples compared to normal aortic tissue. This upregulation in diseased tissue supports that OPN is involved in the structural remodeling and inflammatory activation of the aortic wall. Although the authors did not advocate for OPN as a standalone diagnostic biomarker, they emphasized its relevance in the underlying pathophysiology of aortopathies. The findings point to a potential future role for OPN in the clinical evaluation of chronic or degenerative aortic disease, especially if validated in larger cohorts.

#### 7.10.6. Semaphorin 7A

Semaphorin 7A (Sema7A) is a protein belonging to the semaphorin family, a group of molecules primarily involved in axonal guidance (the orientation of nerve connections) during nervous system development. However, many semaphorins, including Sema7A, also act as important extra-neuronal factors, particularly in immune responses, inflammation, angiogenesis, and tissue repair. A recent clinical study by Lyu et al. [127] has shown that serum levels of Sema7A are significantly elevated in patients with AAD compared to those with AMI or PE or healthy individuals. In a cohort comprising 85 AAD patients, 55 with AMI, 15 with PE, and 30 healthy controls, Sema7A levels were consistently higher in individuals with aortic dissection. Sema7A also showed a positive correlation with D-dimer levels, and its concentration decreased following surgical or endovascular intervention. ROC analysis revealed a diagnostic accuracy, evaluated as an AUROC of 0.842 for Sema7A, compared to 0.788 for D-dimer, suggesting potentially superior sensitivity and specificity in differentiating AAD from other causes of acute chest pain. These findings imply that Sema7A could emerge as a useful and early biomarker to distinguish AAD from other acute cardiovascular conditions.

#### 7.10.7. NOTCH Pathway

The Notch signaling pathway is essential for the structural development and functional maintenance of the aortic wall. It regulates cellular processes such as differentiation, proliferation, and apoptosis in vascular smooth muscle cells (VSMCs), and its activity is closely tied to the mechanical integrity of the vessel wall. Increasing evidence supports a role for dysregulated Notch signaling in the pathogenesis of TAAD. Malashicheva et al. [128] highlighted the importance of this pathway in maintaining aortic wall architecture and enabling the vessel to respond adaptively to biomechanical stress. Disruption of Notch signaling, they argue, contributes to structural weakening of the aortic media and links developmental defects with adult-onset vascular disease. Zou et al. [129] reported hyperactivation of Notch signaling in tissue samples from patients with descending thoracic aortic aneurysms and dissections. Their findings suggest that this overactivation may be a compensatory response to aortic wall injury, promoting VSMC proliferation and extracellular matrix remodeling in an attempt to preserve vessel integrity. Despite these mechanistic insights, the clinical application of Notch-related biomarkers remains unrealized. No circulating markers related to the Notch pathway have been validated for use in acute aortic syndromes, and their diagnostic or prognostic utility in emergency settings has yet to be demonstrated. Nonetheless, the accumulating evidence underscores the biological relevance of Notch signaling in aortic disease and provides a strong rationale for further research. Future studies may clarify whether this pathway could serve as a target for therapy or monitoring in chronic and acute vascular conditions.

#### 7.10.8. Other Emerging Biomarkers

**Figure 2:**
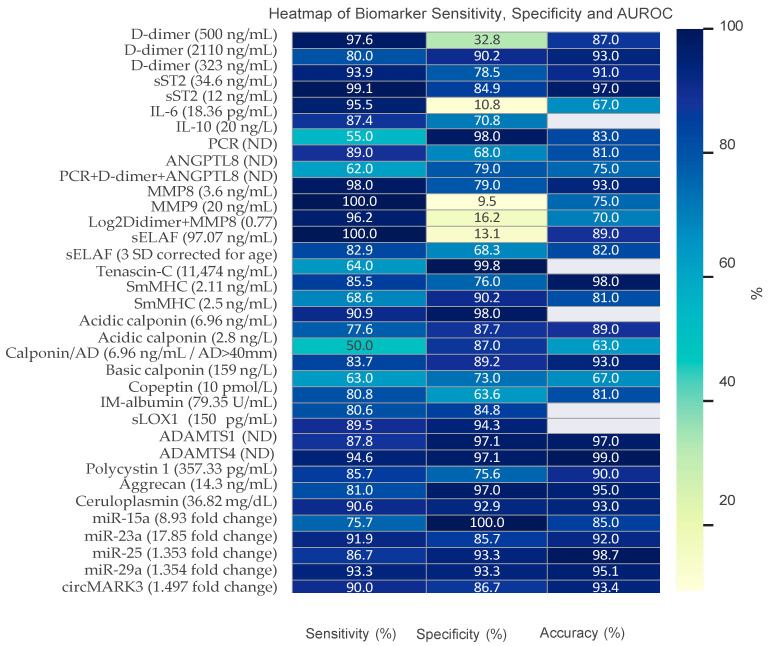
Heatmap of biomarkers with sensitivity, specificity, and accuracy (AUROC).

Several emerging biomarkers have garnered attention in recent years, although their potential use in diagnosing or managing aortic dissections remains uncertain. Ceruloplasmin (CP) is synthesized during the acute inflammatory phase. Elevated CP levels have been associated with various conditions, including heart failure and abdominal aortic aneurysm. Ma et al. demonstrated that CP levels correlate with platelet count and CRP in AD, showing a sensitivity of 90.6% and a specificity of 92.9% at a cutoff of 36.82 mg/dL. Additionally, increased CP levels are linked to a higher risk of false lumen thrombosis [130]. Vinculin is a cytoskeletal protein critical for cell adhesion and structural integrity. Alterations in vinculin contribute to aortic wall stiffness and an increased AAD risk. Wang et al. [131] found elevated vinculin levels in AAD compared to other diseases. Transforming Growth Factor-Beta (TGF-β), a cytokine involved in cellular processes and ECM production, has been studied in AAD. Suzuki et al. [132] showed that TGF-β levels doubled in type-A AAD compared to type-B AD within the first 24 h of symptom onset. Osteoprotegerin (OPG), part of the TNF receptor superfamily, regulates bone and vascular biology. The OPG/TRAIL ratio has been identified as a predictor of 30-day mortality in AAD. A ratio below 4 indicates low risk, while a ratio above 33 signifies high risk [133]. Uric acid, a byproduct of purine metabolism, is elevated in AAD patients and serves as an independent risk factor. Multiple studies find high serum levels but association with AAD severity and prognosis deserves further investigations [134]. In Table 3 and Table 4 and Figure 2, data on the accuracy of biomarkers in the diagnosis of AAS are summarized.

#### 7.10.9. Future Direction

A wide array of biomarkers has been proposed as potentially useful both in facilitating the diagnosis and in predicting the prognosis of patients with AAS. Nevertheless, the available evidence is often preliminary, typically derived from single-center studies involving small sample sizes. In many cases, critical factors such as the time elapsed from symptom onset to biomarker measurement are not accounted for, nor are patients with similar clinical presentations but different final diagnoses adequately considered. Furthermore, especially in the ED setting—where timely diagnosis is essential—laboratory techniques used to assay these biomarkers must be capable of delivering rapid results, ideally within minutes. However, this is not always feasible for all the biomarkers described in the literature. Looking forward, the future of research in this field lies in the integration of clinical scores and multiple biomarkers into composite diagnostic panels. These may be further enhanced by artificial intelligence techniques capable of processing and interpreting complex data sets, ultimately contributing to the development of accurate and efficient diagnostic algorithms for AAS. Moreover, advanced methodologies like proteomics and metabolomics could help to identify novel markers and pathways relevant to AAS. Validation across diverse populations and resource-limited settings, coupled with standardized biomarker assays, will be essential to ensure global relevance and applicability. These integrative strategies have the potential to optimize diagnostic accuracy and improve patient outcomes, particularly in emergency department settings, addressing the urgent need for timely and precise management of AAS.

## 8. Conclusions

Diagnostic scores and biomarkers are useful in the assessment and management of AAS, providing vital guidance for clinicians navigating the complexities of these life-threatening conditions. Tools such as the ADD-RS, with its simplicity, and the more integrative AORTAs score offer structured frameworks for risk stratification and decision-making. Ongoing advancements, particularly the incorporation of biomarkers and advanced imaging, promise to further enhance the accuracy and applicability of these tools. Biomarkers like D-dimer, along with emerging candidates such as sST2 and MMP-9, show significant potential in improving diagnosis and prognosis. However, challenges related to specificity, cohort variability, and assay differences underscore the need for large-scale, multi-center trials to validate their clinical utility.

## Figures and Tables

**Table 1:** AAD anatomical classifications.

**The Bakey System**	
Type 1	The dissection begins in the ascending aorta and involves the aortic arch and descending aorta.
Type 2	The dissection originates in and is confined to the ascending aorta.
Type 3	The dissection starts in the descending aorta and extends distally.
3a	Extends above the diaphragm.
3b	Extends below the diaphragm.
**Stanford classification**	
Type A	Involves the ascending aorta, regardless of the location of the primary intimal tear. It is defined as a dissection occurring proximal to the brachiocephalic artery.
Type B	Originates distal to the left subclavian artery and is confined to the descending aorta. The entry tear is located beyond the origin of the innominate artery [6]

**Table 2:** ADD score, D-dimer. RIPP score and AORTA score in acute aortic syndrome: sensitivity and specificity of single and combined use.

Authors	Cut-Off	Sensitivity % (95% CI)	Specificity % (95% CI)	PPV %	NPV %
Nazerian P. et al. [36]	ADD-RS ≥ 1	95 (91.5–97.4)	26.4 (24.3–28.7)	16.2 (14.3–18.3)	97.3 (95.3–98.6)
Nazerian P. et al. [36]	D-dimer ˃ 0.5 g/L	96.7% (93.6–98.6)	64% (61.6–66.4)	28.7% (25.6–32)	99.2% (98.5–99.7)
Nazerian P. et al. [36]	ADD-RS ˃ 0 + D-dimer ˃ 0.5 g/L	99.6 (97.7–100)	18.2 (16.4–20.2)	15.4 (13.7–17.3)	99.7 (98.1–100)
Nazerian P. et al. [36]	ADD-RS ˃ 1 + D-dimer ˃ 0.5 g/L	98.8 (96.4–99.7)	57.3 (54.9–59.7)	25.8 (23–28.7)	99.7 (99.1–99.9)
Gorla R. et al. [37]	ADD-RS ˃ 0	98.8	64.6	44.9	99.5
Gorla R. et al. [37]	D-dimer ˃ 0.5 g/L	97.6	63.2	43.7	98.9
Gorla R. et al. [37]	ADD-RS = 0 + D-dimer ˃ 0.5 g/L	100	67.5	1.6	100
Gorla R. et al. [37]	ADD-RS 1 + D-dimer ˃ 0.5 g/L	93.5	63.2	21.5	98.9
McLatchie R. et al. [38]	ADD-RS ≥ 1	69 (39–91)	59 (58–60)	40 (18–76)	99.9 (99.7–100)
McLatchie R. et al. [38]	D-dimer ˃ 0.5 g/L	57 (18–90)	61 (57–65)	1.9 (0.6–4.2)	99.8 (99.6–99.9)
McLatchie R. et al. [38]	ADD-RS 1 + D-dimer ˃ 0.5 g/L	100 (69–100)	8 (7–9)	42 (2–77)	100 (98.2–100)
Deng L. et al. [39]	ADD-RS ˃ 1 + D-dimer ˃ 2.0 g/L	93.1	70.2	34.6	98.4
Kotani Y. et al. [40]	ADD-RS ˃ 1 + positive age adjusted d-dimer	88.0 (68–97)	45.0 (30–60)	-	-
Ren S. et al. [41]	ADD RS > 0 + D-dimer > 0.5 g/L	99.8 (98.7–100)	21.8 (12.1–32.6)	-	-
Ren S. et al. [41]	ADD RS > 1 + D-dimer > 0.5 g/L	98.3 (94.9–99.5)	51.4 (38.7–64.1)	-	-
Ohle R. [42]	RIPP ≥ 2	99.7 (98.5–99.9)	53.0 (47.9–58.1)	68.0 (64.0–71.7)	99.5 (97.2–99.9)
**Authors**	**Cut-Off**	**Sensitivity % (95% CI)**	**Specificity % (95% CI)**	**LR+ (95% CI)**	**LR− (95% CI)**
Morello F. [43]	High prevalence population AORTAs ≤ 1/D-Dimer age adjusted	98.3 (95.7–99.5)	30 (26.9–33.4)	1.41 (1.34–1.47)	0.06 (0.02–0.15)
Morello F. [43]	High prevalence population AORTAs ≤ 1/D-Dimer 0.5 g/L	99.1 (96.9–99.9)	30.2 (27–33.5)	1.42 (1.35–1.49)	0.03 (0.01–0.11)
Morello F. [43]	Low prevalence population AORTAs ≤ 1/D-Dimer age adjusted	100 (92.7–100)	48.7 (43.8–53.7)	1.95 (1.74–2.14)	0 (0–0.12)
Morello F. [43]	Low prevalence population AORTAs ≤ 1/D-Dimer 0.5 g/L	98 (89.3–99.6)	52.8 (47.9–57.7)	2.08 (1.86–2.33)	0.04 (0–0.14)

**Table 3:** Sensibility, sensitivity, and accuracy evaluated as area under the ROC (AUROC) curves of biomarkers for the diagnosis of acuta aortic dissection.

Authors	Biomarker	Cut-Off	Sensitivity % (95% CI)	Specificity % (95% CI)	Accuracy (AUROC)
Giachino et al., 2013 [77]	D-dimer	500 ng/mL	97.6 (87.4–99.9)	32.8 (21.3–46.0)	0.87 (0.08–0.97)
Meng et al., 2019 [135]	D-dimer	500 ng/mL	100	51.3	ND
Morello et al., 2018 [93]	D-dimer	500 ng/mL	95.2 (88.3–98.1)	65.4 (58.5–71.8)	0.92 (0.89–0.96)
Morello et al., 2020 [60]	D-dimer	500 ng/mL	95.8 (88.1–99.1)	30.7 (19.6–43.7)	0.84 (0.75–0.91)
Peng et al., 2015 [68]	D-dimer	2.11 µg/mL	80	90.2	0.93 (0.87–0.98)
Wang et al., 2017 [59]	D-dimer	323 ng/mL	93.9	78.5	0.91 (0.88–0.94)
Zhang et al., 2023 [75]	D-dimer	500 ng/mL	74 (69–79)	76 (72–80)	0.82 (0.79–0.85)
Suzuki I et al., 2009 [56]	D-dimer	500 ng/mL	96.6 (90.3–99.3)	46.6 (37.9–55.5)	0.84 (0.78–0.89)
Sodek G et al., 2008 [89]	D-dimer	500 ng/mL	97.0 (94–98)	59 (53–64)	0.94
Wang et al., 2017 [59]	sST2	34.6 ng/mL	99.1 (ND)	84.9	0.97 (0.95–0.98)
Yang et al., 2020 [72]	D-dimer	ND	67	98	0.88 (0.8–0.93)
Morello et al., 2020 [60]	sST2	12 ng/mL	95.5 (88.8–98.7)	10.8 (4.8–20.2)	0.67 (0.61–0.74)
Qu et al., 2015 [63]	IL-6	18.36 pg/mL	87.4	70.8	ND
Forrer a et al., 2021 [65]	IL-10	20 ng/L	55	98	0.83 (0.72–0.94)
Yang et al., 2020 [72]	PCR	ND	89	68	0.81 (0.73–0.88)
Yang et al., 2020 [72]	ANGPTL8	ND	62	79	0.75 (0.66–0.83)
Yang et al., 2020 [72]	PCR + D-dimer + ANGPTL8	ND	98	79	0.93 (0.88–0.97)
Giachino et al., 2013 [77]	MMP8	3.6 ng/mL	100 (93.2–100)	9.5 (3.9–18.5)	0.75
Giachino et al., 2013 [77]	MMP9	20 ng/mL	96.2 (86.8–99.5)	16.2 (87.26.6)	0.70
Giachino et al., 2013 [77]	Log2Didimer + MMP8	0.77	100 (91.6–100)	13.1 (5.8–24.2)	0.89 (0.82–0.95)
Peng et al., 2015 [68]	sELAF	97.07 ng/mL	82.9	68.3	0.82 (0.73–0.91)
Shinohara et al., 2003 [79]	sELAF	3 SD corrected for age	64	99.8	ND
Ma et al., 2024 [136]	Tenascin-C	11474 ng/mL	85.5	76.0	0.98 (0.96–0.99)
Peng et al., 2015 [68]	SmMHC	2.11 ng/mL	68.6	90.2	0.81 (0.71–0.91)
Suzuki et al., 2000 [84]	SmMHC	2.5 ng/mL	90.9	98	ND
Suzuki et al., 2008 [85]	SmMHC	2.5 ng/mL	90	97	ND
Lian et al., 2023 [86]	Acidic calponin	6.96 ng/mL	77.6	87.7	0.89
Suzuki et al., 2008 [85]	Acidic calponin	2.8 ng/L	50	87	0.63
Lian et al., 2023 [86]	Calponin and aortic diameter	6.96 ng/mL AD > 40 mm	83.7	89.2	0.93
Suzuki et al., 2008 [85]	Basic calponin	159 ng/L	63	73	0.67
Morello et al., 2018 [93]	Copeptin	10 pmol/L	80.8 (72.2–87.2)	63.6 (56.9–69.9)	0.81 (0.75–0.86)
Yang et al., 2019 [95]	IM-albumin	79.35 U/mL	80.6	84.8	ND
Kobayashi et al., 2013 [96]	sLOX1	150 pg/mL	89.5	94.3	ND
Li et al., 2017 [119]	ADAMTS1	ND	87.8	97.1	0.97 (0.94–0.99)
Li et al., 2017 [119]	ADAMTS4	ND	94.6	97.1	0.99 (0.97–1.00)
Lyu et al., 2023 [127]	Sema7A	ND	ND	ND	0.84 (0.78, 0.91)
Peng W et al., 2015 [68]	Polycystin 1	357.33 pg/mL	85.7	75.6	0.90 (0.83–0.96)
Konig CK et al., 2021 [116]	Aggrecan	14.3 ng/mL	81	97	0.95
Ma et al., 2021 [130]	Ceruloplasmin	36.82 mg/dL	90.6	92.9	0.93 (0.88–0.97)
Dong et al., 2017 [108]	miR-15a	8.93 (fold change)	75.7	100	0.85 (0.85–0.96)
Dong et al., 2017 [108]	miR-23a	17.85 (fold change)	91.	85.7	0.92 (0.85–0.99)
Xu et al., 2017 [109]	miR-25	25 1.353 (fold change)	86.7	93.3	0.987 (0.937–1.000)
Xu et al., 2017 [109]	miR-29a	1.354 (fold change)	93.3	93.3	0.951 (0.86–1.00)
Tian et al., 2019 [111]	circMARK3	1.497 (fold change)	90.0	86.7	0.934 (0.87–1.0)

**Table 4:** Comparative evaluation of emerging and established biomarkers for acute aortic syndromes.

Biomarker	Clinical Rationale (Potential Utility)	Evidence, Size, Heterogeneity	Data Robustness	Clinical Adoption Potential	Limitation	Feasibility	Recommended Current Role
D-dimer	Fibrinolysis marker; helps rule-out AAS combined with low clinical probability	Large multicenter; mixed designs but consistent sensitivity within 24 h	Moderate/ High	High	Low specificity; false negatives possible)	Community ED, secondary and tertiary centers	Clinical use
sST2	Myocardial stretch/inflammation	Small–moderate; heterogeneous cut-offs	Low	Low	Limited availability; non-specific	Tertiary	Research
IL-6	Systemic inflammationmay track extent/complication	Small–moderate; variable timing assays	Low–Moderate	Low	Non-specific; slow TAT	Tertiary	Research
IL-10	Anti-inflammatory response	Small; heterogeneous	Low	Low	Limited incremental value)	Tertiary	Research
CRP	Acute-phase, prognostic interest only	Large literature but non-specific	Low	Very low	Poor specificity	Community ED	Abandon
ANGPTL8	Lipid/inflammation axis	Very small, exploratory	Very low	Low	Lack of esternal validation	Tertiary	Research
MMP-8	ECM degradation	Small; assay heterogeneity	Low-moderate	Low	Limited rapid test	Tertiary	Research
MMP-9	ECM remodeling	Small–moderate; conflicting thresholds	Low	Low	Non-specific; timing effects)	Tertiary	Research
Copeptin	Released in response to stess	Small, single centre study	Low	Low	Non-specific nor sensitive	Tertiary	Abandon
sELAF	Elastin degradation	Small; assay variability	Low	Low	Lack of standardization	Tertiary	Research
Tenascin-C	ECM stress protein	Small–moderate	Low-moderate	Low	Limited assays	Tertiary	Research
smMHC	Smooth-muscle injury; early release	Small–moderate; promising	Moderate	Low-moderate	Short diagnostic window	Tertiary	Research
Acid Calponin	SMC injury	Small; exploratory	Low	Low	Limited availability	Tertiary	Research
Basic Calponin	SMC injury	Small; exploratory	Low	Low	Limited availability	Tertiary	Research
IMA	Global ischemia marker	Small	Low	Low	Poor specificity	Tertiary	Abandon
sLOX-1	Endothelial injury/oxidized LDL receptor	Small–moderate	Moderate	Low-moderate	Limited availibility	Tertiary	Research
ADAMTS-1	Protease, ECM turnover	Small	Low	Low	Only preliminary data	Tertiary	Research
ADAMTS-4	Protease, ECM turnover	Small	Low	Low	Only preliminary data	Tertiary	Research
Sema7A	Immune/semaphorin pathway	Very small	Very low	Very low	preliminary data	Tertiary	Research
Polycystin-1	Structural protein	Minimal/indirect	Very low	Very low	preliminary data	Tertiary	Abandon
Aggrecan	ProteoglycanECM damage	Very small	Very low	Very low	Limited specificity	Tertiary	Research
Ceruloplasmin	Acute-phase/oxidative	Large	Very low	Very low	nonspecific	Tertiary	Abandon
miR-15a	Tissue injury signature	Small cohorts	Low	Low	Preliminary data, PCR-based; slow TAT	Tertiary	Research
miR-23a	Tissue injury signature	Small cohorts	Low	Low	Preliminary data, PCR-based; slow TAT	Tertiary	Research
miR-29a	ECM remodeling	Small cohorts	Low	Low	Preliminary data, PCR-based; slow TAT	Tertiary	Research
circMARK3	Omics signal	Small cohorts, heterogeneous	Low	Low	Preliminary data	Tertiary	Research

Legend. AAS: acute aortic syndrome; ECM: extracellular matrix; SMC: smooth muscle cell; IMA: ischemia-modified albumin; LDL: low-density lipoprotein; IL: interleukin; TAT: turnaround time; miR: microRNA; circ: circular RNA; sELAF: soluble elastin fragment; MMP: matrix metalloproteinase; smMHC: smooth-muscle myosin heavy chain.
